# Direct Ink 3D Printing of Porous Carbon Monoliths for Gas Separations

**DOI:** 10.3390/molecules27175653

**Published:** 2022-09-02

**Authors:** Marisa L. Comroe, Kurt W. Kolasinski, Dipendu Saha

**Affiliations:** 1 One Chemical Engineering Department, Widener University, 1 University Place, Chester, PA 19013, USA; 2 Department of Chemistry, West Chester University, West Chester, PA 19383, USA

**Keywords:** porous carbon, 3D printing, gas separations

## Abstract

Additive manufacturing or 3D printing is the advanced method of manufacturing monolithic adsorbent materials. Unlike beads or pellets, 3D monolithic adsorbents possess the advantages of widespread structural varieties, low heat and mass transfer resistance, and low channeling of fluids. Despite a large volume of research on 3D printing of adsorbents having been reported, such studies on porous carbons are highly limited. In this work, we have reported direct ink 3D printing of porous carbon; the ink consisted of commercial activated carbon, a gel of poly(4-vinylphenol) and Pluronic F127 as plasticizer, and bentonite as the binder. The 3D printing was performed in a commercial 3D printer that has been extensively modified in the lab. Upon 3D printing and carbonization, the resultant 3D printed porous carbon demonstrated a stable structure with a BET area of 400 m^2^/g and a total pore volume of 0.27 cm^3^/g. The isotherms of six pure-component gases, CO_2_, CH_4_, C_2_H_6_, N_2_, CO, and H_2_, were measured on this carbon monolith at 298 K and pressure up to 1 bar. The selectivity of four gas pairs, C_2_H_6_/CH_4_, CH_4_/N_2_, CO/H_2_, and CO_2_/N_2_, was calculated by Ideally Adsorbed Solution Theory (IAST) and reported. Ten continuous cycles of adsorption and desorption of CO_2_ on this carbon confirmed no loss of working capacity of the adsorbent.

## 1. Introduction

Porous carbon-based materials are widely used in many types of industries and household applications [1]. Porous carbon is extensively used in the field of water purification [2], air purification [3], gas separation [4] and storage [5], catalysis and catalyst support [6], electrode materials [7], supercapacitors [8], batteries [9], gas masks [10], medical applications [11], and many more. Porous carbon is available in many different forms, names, and properties, such as activated carbon or charcoal, mesoporous carbon, carbon molecule sieve (CMS), activated carbon fiber (ACF), and others. The key attractive features of porous carbons are inexpensive and sustainable precursors, tunability of porosity, structural stability in various environments, and affinity to a wide range of molecules to facilitate adsorption behaviors. In the majority of applications, carbon materials are employed as powdered forms, pellets, or beads. In the case of application of carbon in powdered form, it possesses the difficulty of large pressure drop, handling problems, and finding suitable supports. In order to overcome these hurdles, structured forms of carbons were needed, and in order to meet such demand, pelletized forms of carbons have been employed. As of today, it is the most state-of-the-art form of carbon to be used in all industrial setups. However, the pelletized form of carbon possesses a few drawbacks, including high mass and mass transfer resistance, high-pressure drop, fluidization, and unwanted channeling or maldistribution of the flowing fluids [12,13]. Different types of customized formulations also exist for fabricating structured adsorbents, such as surface extrusion, direct deposition, or matrix incorporation [14,15,16]; however, the majority of those structures do not possess the suitability to incorporate porous carbon-based materials.

In modern times, additive manufacturing or 3D printing of adsorbents has evolved that facilitates the production of on-demand shape and size of the adsorbent monoliths, which overcome most of the drawbacks mentioned above [17,18]. The 3D printing of adsorbents can be achieved by different techniques, such as direct ink 3D printing [19], secondary seeding growth [20], polymer phase separation [21], sol-gel printing, or binderless printing [22]. While the published literature on 3D printed structured adsorbents, including different types of 3D printed carbon composites [23] or graphene-based materials [24,25], is quite large, the research on 3D printed structures and monolithic porous carbon adsorbents is very limited. Direct ink 3D printing of carbon aerogels was reported for resorcinol-formaldehyde (RF) resins that were carbonized to produce a carbon aerogel. This was later activated with CO_2_ to produce porous carbon with a high BET area of 2000 m^2^/g [26]. The 3Dprinted and hard-templated mesoporous carbon was reported with SiO_2_ as templating agent and starch/gelatin as the carbon precursor. Several steps were followed after 3D printing, including freeze-drying, carbonization, and template removal to obtain the final form of the porous carbon monolith. The BET surface area varied from 183 to 833 m^2^/g [27,28]. UV-curable clear 2005T resin from Miicraft was employed as a carbon precursor to print the polymer in a DLP (digital light processing) 3D printer, and the resultant structure was carbonized in order to obtain the carbon [29]. The BET area of this carbon was not reported, however, owing to lack of porogen or activation, it is expected that the surface area will be very small. The 3D printed electrode of the supercapacitor was fabricated by printing a mixture of activated carbon, polyvinyl alcohol (PVA), and H_3_PO_4_ [30]. The maximum amount of activated carbon was limited to 35 wt.% only. Although the pristine BET area was as high as 904 m^2^/g, it is expected that the available surface area in the electrode will be much smaller, owing to the small percentage of carbon. Nonetheless, 3D printing was performed with a lithography-based technique by incorporating ZIF-8 as a templating agent and a mixture of UV-curable polymers [31]. The carbon was synthesized by pyrolyzing the composite and dissolving the template. Therefore, no direct attempt was made to 3D print the carbon itself.

In a unique approach, lithographic 3D printing was achieved by employing photoinduced copolymerization of pentaerythitol tetraacrylate and divinylbenzene as the carbon precursor alkylphthalate as soft-template or porogen [32]. The BET surface area was 64 to 125 m^2^/g; however, additional CO_2_ activation increased the BET area up to 3019 m^2^/g.

Therefore, 3D printing of porous carbon-based materials is very limited compared to that of other adsorbents. In the majority of cases, 3D printing required either several steps [27,28], resulting in low porosity [29,30] in the final 3D printed structure, or very specialized polymer as carbon precursor [33]. Therefore, there is a need to develop a simplified approach for direct ink 3D printing of porous carbons with moderate to high surface area. It is also highly advantageous to incorporate commercially available porous or activated carbon as one of the constituents of 3D printed structure. Therefore, in order to address those aspects in this research, we have reported a methodology of direct ink 3D printing of porous carbons with the ink consisting of commercial activated carbon, poly(4-vinylphenol)-plutonic F127 gel as a plasticizer, and bentonite as the binder. The 3D printed structure was carbonized to obtain the final form of porous carbon, where resorcinol-formaldehyde-plutonic F127 yielded additional porosity to the carbon. The 3D printed porous carbon monolith was used for demonstrating gas-separation applications.

## 2. Experimental Section

### 2.1. Synthesis of Ink for 3D Printing

**Figure 1:**
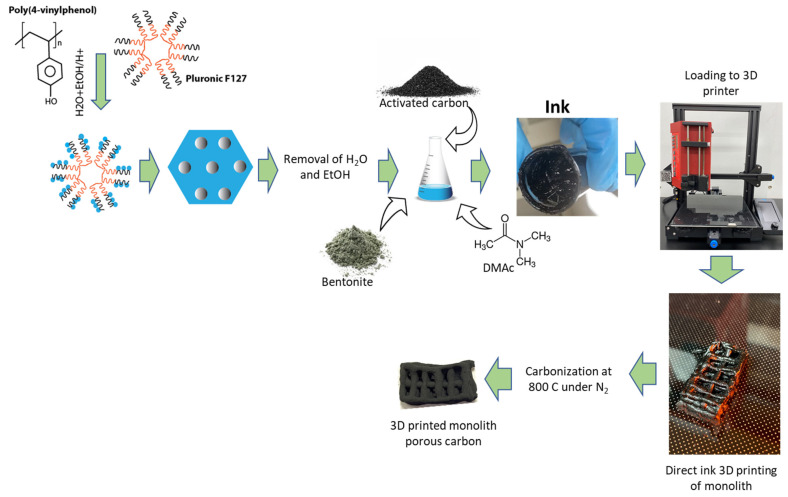
Schematic of synthesis of ink for direct ink 3D printing.

The commercial activated carbon that was chosen for this research is granular activated carbon from Calgon with a BET specific surface area of 831 m^2^/g and a total pore volume of 0.47 cm^3^/g. This carbon was ground in a coffee grinder to form a fine powder. After that, 12 g poly(4-vinylphenol) and 6 g Pluronic F127 were dissolved in an 18 mL mixture of deionized (DI) water and ethanol (1:1 *v*/*v*) and 3 mL HCl (36%). After stirring for a day, a polymer mixture settled at the bottom. The top layer consisting of mostly solvents was discarded, and the polymer layer was added with 12 mL N N-dimethylacetamide (DMAc). The polymer layer was completely dissolved into it. Then, 24 g of previously ground activated carbon mixture was added to the mixture in small increments of 1 g. In the same mixture, a total of 7.5 g of bentonite was added in increments of 0.5 g. The mixture was vigorously stirred with a glass rod until a homogeneous mixture was obtained. The schematic of ink production is shown in Figure 1.

### 2.2. Modification of 3D Printer and Fabrication of 3D Printed Structure

**Figure 2:**
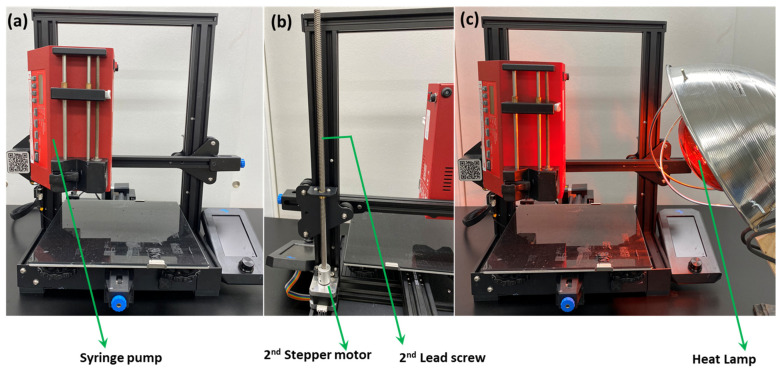
Different types of modifications of 3D printer.

The 3D printer that was used in the course of this research is the Ender 3 v2. After proper installation and construction of the printer, it underwent a few custom modifications to ensure the printing of the carbon monolith. The heated nozzle of the original 3D printer was replaced by a syringe pump (New Era), which was attached to the printer with screws (Figure 2). The syringe pump was loaded with a 3 mL syringe fitted with an 18-gauge dispensing needle. The weight of the syringe pump posed an instability and a wobbling motion in the stepper motor, which allowed the printing head (now replaced with the syringe pump) to move in the z-direction. In order to avoid instability, a 2nd stepper motor along with a 2nd lead screw was added to the printer (Figure 2b). This arrangement allowed more stability as the syringe pump moved in all three directions and compensated for the added weight of the pump.

In order to correlate the speed of the syringe pump and the printing speed (as those two parameters are independently set; the printing speed was set by the software and the flow rate in the syringe pump was set manually) the printer speed (mm/min) was converted into the syringe pump speed (mL/s) followed by necessary adjustments in real-time if there was any noticeable speed difference was observed during the printing.

Finally, it was observed that ease of printing can be achieved at a particular rheology of ink that does not allow it to dry quickly after printing, resulting in partial mixing of printed layers. In order to avoid this problem, an additional heat lamp was set in the proximity of the printing area (Figure 2c) that helped to dry ink rapidly, thereby preventing interlayer mixing.

### 2.3. The 3D printing of Carbon Monolith

Upon modifying the 3D printer, about 2.5 mL of ink was loaded onto a disposable syringe and fitted with an 18-gauge dispenser (blunt) needle. The syringe assembly was loaded onto the syringe pump attached with the modified 3D printer. The flow rate from the syringe was set as 0.6 mL/min while printing the structure. Over the course of printing, the heat lamp was turned on in order to quickly dry the ink. Upon completion of printing, the 3D printed structure was scraped out from the plate. After that, the monolith was put into a porcelain boat, and the boat was inserted into the Lindberg-blue^TM^ tube furnace for carbonization. The furnace was heated to 900 °C at the ramp rate of 10 °C/min and cooled down to room temperature. All the heating and cooling profiles were performed under N_2_ gas flow.

### 2.4. Characterization of Ink and 3D-Printed Carbon Monolith

The ink was characterized by thermogravimetric analysis (TGA) in TA instruments’ DCA Q600 thermogravimetric analyzer under N_2_ and air. The carbonized monolith was characterized by optical images, pore textural properties, and scanning electron imaging (SEM). The pore textural properties, including BET specific area, pore volume, and pore size distribution, were calculated from N_2_ adsorption–desorption analysis at 77 K and CO_2_ adsorption at 298 K, which were performed in the Autosorb-iQ-Any gas instrument (Quantachrome, Boynton Beach, FL, USA). The SEM images were captured by the FEI Quanta 400 (Thermo Fisher Scientific, Hillsboro, OR, USA) in secondary electron mode. X-ray diffraction patterns were obtained by the Miniflex XRD instrument (Rigaku, Austin, TX, USA). In order to capture the XRD pattern of the 3D printed structure, it was ground to a fine powder in mortar and pestle and introduced within the sample holder.

### 2.5. Gas Adsorption Studies

The gas adsorption isotherms, including CO_2_, CH_4_, C_2_H_6_, N_2_, CO, and H_2_, were measured in the same Autosorb-iQ-Any gas instrument. All the gases were of ultra-high purity (UHP) and obtained from commercial sources (Air Gas). The isotherms were measured at a temperature of 298 K and pressure up to 1 bar. The temperature of the isotherm bath was maintained by an external circulating chiller (Julabo). The cyclability of CO_2_ adsorption and desorption was carried out under the hysteresis mode of operation of the same instrument.

## 3. Results and Discussions

### 3.1. Thermogravimetric Analysis (TGA) of the Ink

**Figure 3:**
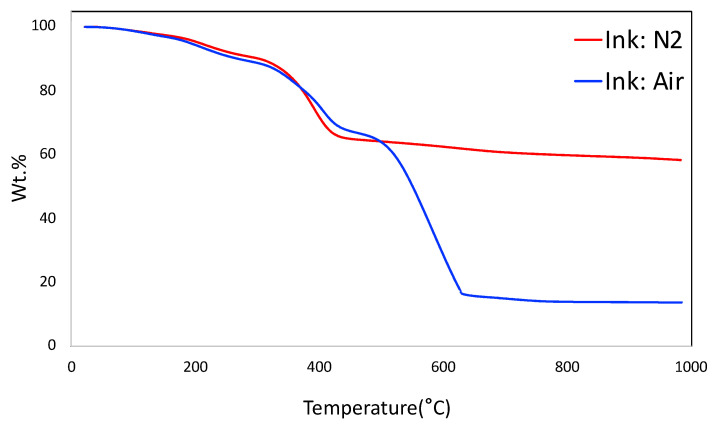
Thermogravimetric analysis (TGA) of ink under N_2_ and air.

The thermogravimetric analysis (TGA) of the ink under N_2_ and air are shown in Figure 3. The TGA plot under N_2_ simulates the carbonization of the monolith. It shows the prominent weight loss around 400 °C that corresponds to the decomposition of sacrificial F127 from the system, along with a part of decomposition of poly(4-vinylphenol) that occurs in the region of 350–500 °C. It is observed that the total yield of the monolith is around 58.3 wt.%. The TGA plot under air shows that the carbon-based materials were completely burned out at 635 °C. The final yield under air at around 15.9 wt.% corresponds to bentonite in the system.

### 3.2. Characteristics of 3D Printed Porous Carbon Monolith

**Figure 4:**
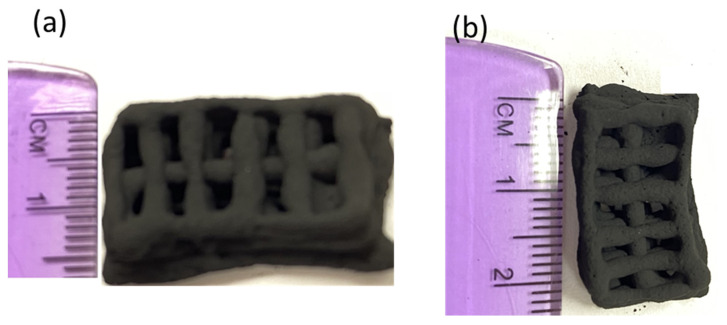
Optical image of 3D printed porous carbon monolith (**a**,**b**).

**Figure 5:**
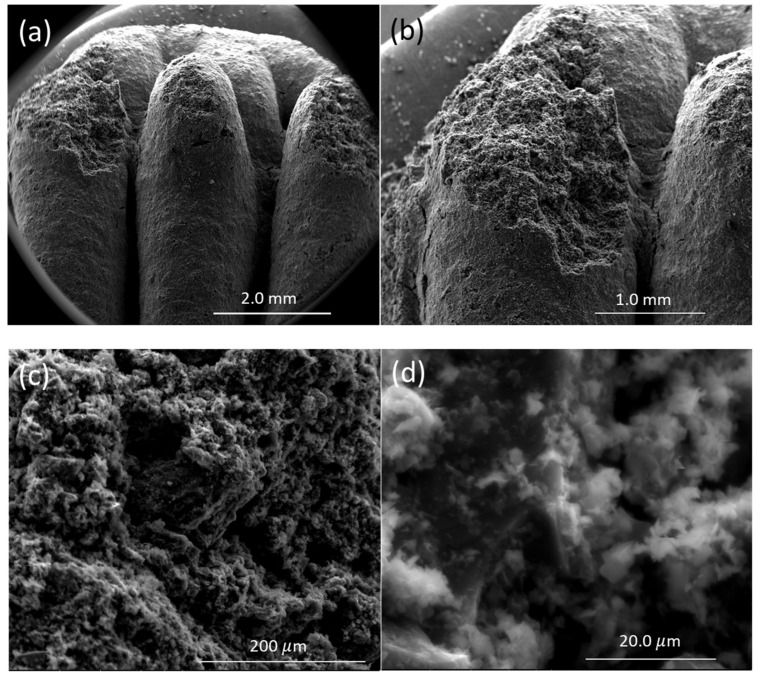
SEM images of 3D printed monolith magnifying the same spot in different levels of magnification: (**a**) 21×, (**b**) 40×, (**c**) 300×, and (**d**) 2400×.

The optical image of the 3D-printed monolith is shown in Figure 4a,b. The length, width, and depth of the individual monolith are around 2.4 cm × 1.4 cm × 0.4 cm. The width of the solid boundary along the mesh is around 2 mm. It is observed that there are some non-uniform widths of the solid boundary that might have been caused by the fluctuations in fluid flow from the syringe in the course of printing. The current three-dimensional structure was chosen to ensure that the monolith could be dried properly before different layers of the structure are mixed with each other, resulting in the collapse of the structure. A rectangular prism in a mesh pattern with a high aspect ratio allowed for a stable print. This is because the rectangle was longer in length than height, which decreased the drying time of one layer before the next layer was printed on top of it. This thereby prevented the layers from sinking into each other. The other limitation associated with the dimension of this particular shape was attributed to the maximum amount of ink that could be held in the syringe (3 mL) and in the course of 3D printing. The SEM images confirmed that the outer surface is relatively smooth with the inner surface exhibiting roughnesses. The SEM images of the monolith are shown in Figure 5a–d. The inner surface has no specific shape or morphology. Most likely, the bright crystalline entities in Figure 5d are the bentonite crystals mixed with carbon matrix.

**Figure 6:**
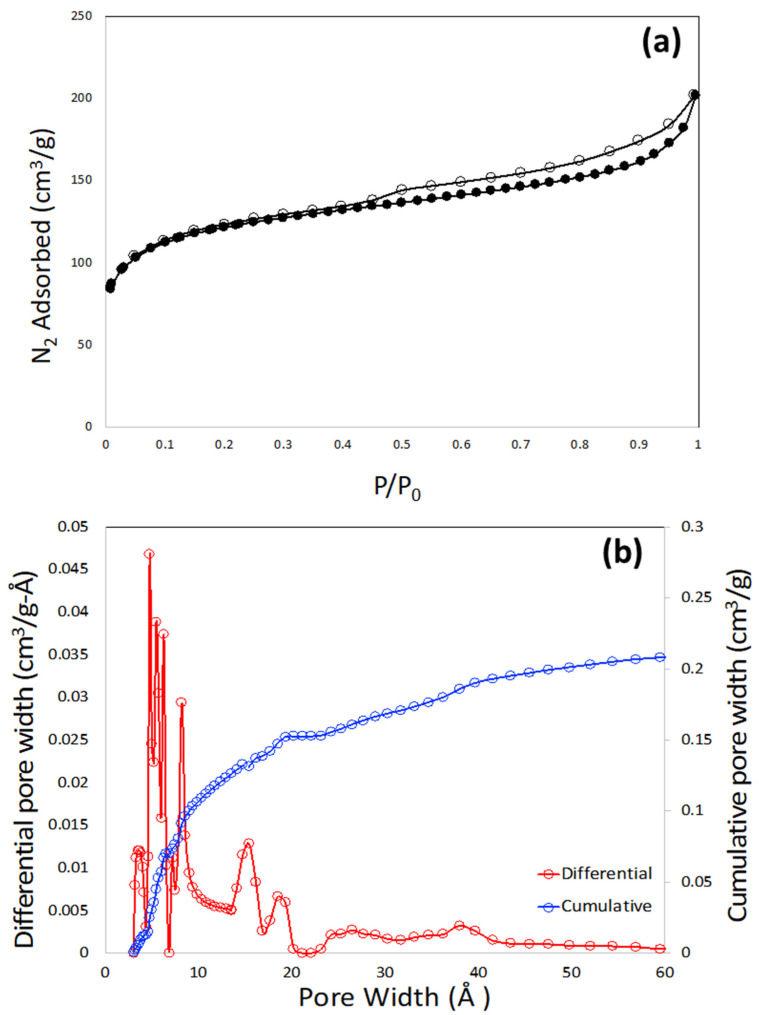
N_2_ adsorption-desorption plot at 77 K (**a**) and pore size distribution plot (**b**) of 3D printed porous carbon monolith.

The pore textural properties of the porous carbon monolith are shown in Figure 6a,b. Figure 6a shows the N_2_ adsorption–desorption plot at 77 K. It is observed that the nitrogen isotherm does not resemble a single type of isotherm according to the IUPAC classifications. The sharp rise in the low (near-zero) pressure region is attributed to the presence of microporosity. The gradual increase in the elevation of the plateau at higher pressure, along with the presence of the hysteresis loop, signifies the presence of mesoporosity. Most likely, this isotherm is closely associated with the combination of type I and type IV isotherm. The type of the hysteresis loop is closely associated with the H4-type of hysteresis [33]. This type of hysteresis is generally caused by mono- and multi-layer adsorption and capillary condensation. The BET specific surface area calculated from the N_2_ adsorption plot is 400 m^2^/g. The pore size distribution was calculated by non-local density function theory (NLDFT) within the built-in software of the instrument, and is shown in Figure 6b.

It needs to be noted that the polymer mixture of poly(4-vinylphenol) and Pluronic F127 is the precursor of so-called soft-templated mesoporous carbon, where poly(4-vinylphenol) acts as the carbon precursor and Pluronic F127 acts as porogen or soft-template. In soft-templating strategy, cross-linked phenolic polymer, mostly from phenol, resorcinol, and phloroglucinol serves as the precursor whereas the surfactant (F127) acts as porogen [34,35]. The width and shape of the mesopore depend on the surfactant molecule leading to the formation of micelles that are hydrogen bonded with phenolic polymer, pH of the synthesis medium, and other processing conditions of the polymer mixture before carbonization. Although a sharp and distinct mesopore peak is observed in most of the soft-templated mesoporous carbons, no such mesopores are observed in this monolith (Figure 6b). Dissolution of the polymer mixture of poly(4-vinylphenol) and Pluronic F127 by DMAc in the course of making the ink probably ruptured the micelles of the surfactant, leading to the disrupted and distributed mesoporosity in the region of 25–60 Å (Figure 6b). The pore size distribution plot also suggested that the monolith has micropore widths in the regions of 19, 15, 8, and 5 Å. The total pore volume of the monolith is 0.27 cm^3^/g.

**Figure 7:**
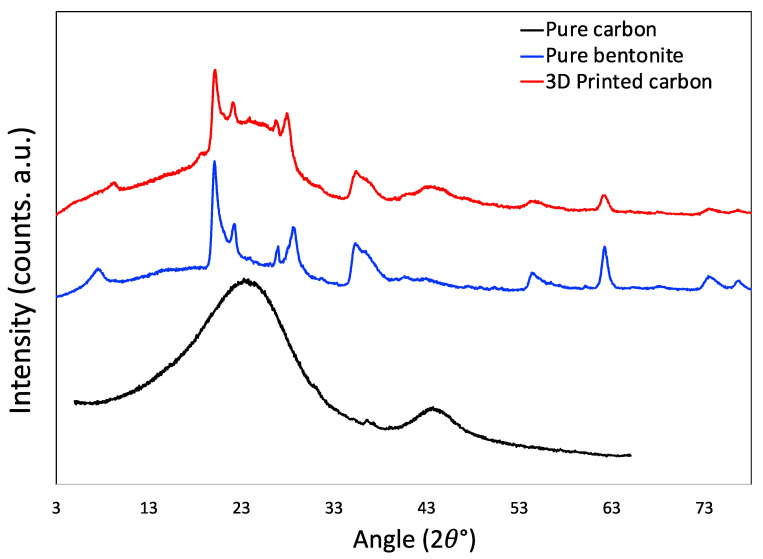
X-ray diffraction patterns of 3D printed carbon monolith, pristine bentonite, and pristine porous carbon without bentonite.

The XRD image is shown in Figure 7. The XRD pattern of pure carbon as control is obtained by grinding the material that was obtained by carbonizing the ink without bentonite powder, i.e., without 3D printing. The XRD of this control carbon material is typical of the other carbon materials, it has two broad peaks at around 23° and 43° that are the remnants of graphitic ordering, which is very common for sp^2^-hybridized carbons. Pure bentonite has several peaks spread along the spectrum that are associated with its crystallinity. The prominent peaks in the bentonite structure are present in 7.7°, 20.1°, 22.3°, 26.9°, 28.7°, 35.6°, 54.6°, and 62.3° positions. In the 3D printed carbon, the presence of all of these peaks is clearly visible, owing to the presence of bentonite in it. The little ‘hump-like’ morphology around 23° and 43° positions still bear the signatures of the original carbons.

### 3.3. Gas Adsorption Studies

**Figure 8:**
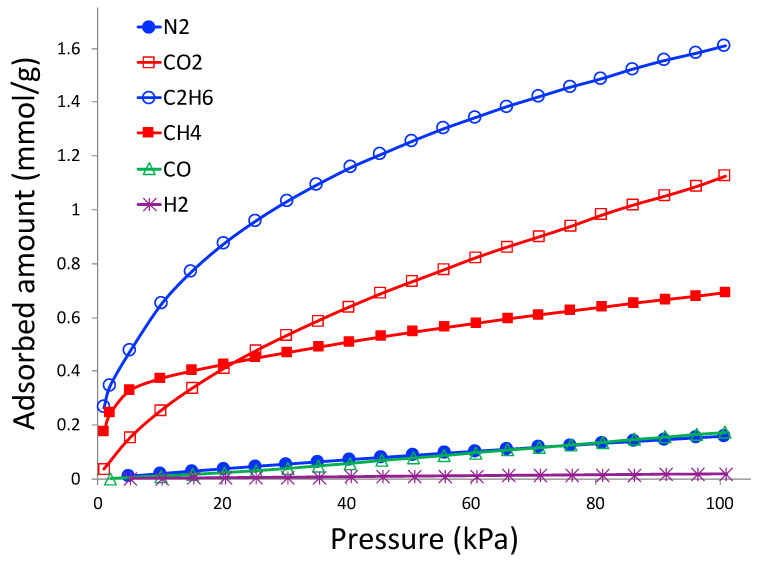
Gas adsorption isotherms on 3D printed porous carbon monolith at 298 K.

Gas adsorption isotherms from CO_2_, CH_4_, C_2_H_6_, N_2_, CO, and H_2_ at 298 K and pressure up to 760 torr are shown in Figure 8. These gas pairs are selected according to industrial needs. In order to minimize greenhouse gas emissions to the atmosphere, CO_2_ needs to be separated from N_2_ from flue gas in coal-powered electricity generation plants, and adsorption plays a crucial role in this separation [36]. Natural gas (CH_4_) needs to be separated from C_2_H_6_ and N_2_ in order to purify and enrich the natural gas [37]. C_2_H_6_ can be recovered and enriched as fuel or as precursor to synthesize other chemicals. CO and H_2_ are constituents of syngas reactions, and adsorption is considered to be one of the feasible ways to separate them [38].

As observed in Figure 8, the highest adsorbed gas is C_2_H_6_, followed by CO_2_ and CH_4_. The lowest adsorbed amount belongs to H_2_; CO and N_2_ have similar adsorption amounts, which are slightly higher than that of H_2_. It is clear that the adsorbent is selective to CO_2_, CH_4_, and CO compared to N_2_, and C_2_H_6_ compared to CH_4_.

All the gas adsorption isotherms are modeled with the Sips equation, which is an empirical form and constructed with the correlations from the Langmuir and Freundlich equations. The Sips equation fits well with most experimental isotherms and can be written as:

(1) $$q=\frac{a_{m}bp^{1/n}}{1+bp^{1/n}},$$

where q and p are adsorbed amount and pressure, respectively. $a_{m}$, b , and n are constants and found by the solver function of Microsoft Excel. The values of the constants are given in Table 1.

As mixed-component adsorption is very challenging to perform, owing to complexity in instrumentation, it is a common practice to perform the pure component adsorption (like that in Figure 8) and report the selectivity of the preferred gas compared to that of the unpreferred one. The equilibrium selectivity ($\alpha_{1/2}$) of component 1 (preferred adsorbate) over component 2 (unpreferred adsorbate) is defined as [39]:

(2) $$\alpha_{1/2}=\frac{x_{1}/y_{1}}{x_{2}/y_{2}}$$

where x and y are the mole fractions of adsorbate in the adsorbed phase and the bulk gas phase, respectively. The most common and popular way of calculating selectivity from adsorption isotherms is the Ideally Adsorbed Solution Theory (IAST), originally proposed by Myers and Prausnitz [40].

**Figure 9:**
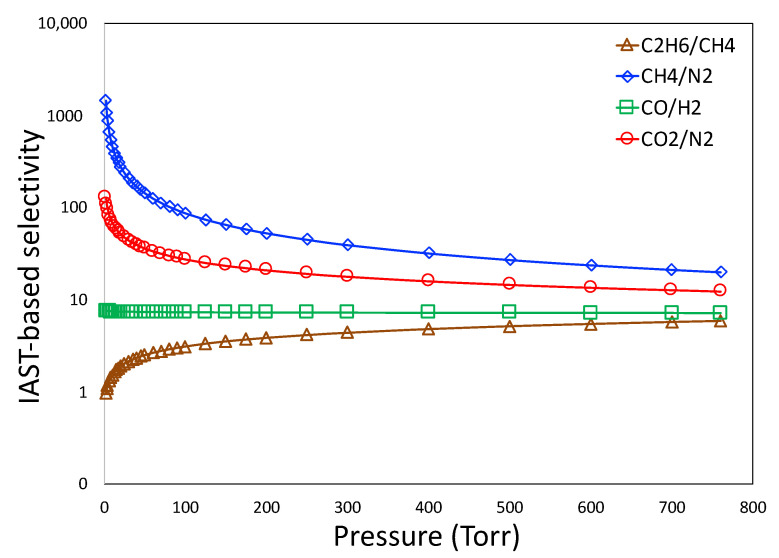
IAST-based selectivity for gas pairs.

The IAST-based selectivity of C_2_H_6_/CH_4_, CH_4_/N_2_, CO/H_2_, and CO_2_/N_2_ as a function of partial pressure are shown in Figure 9. The adsorbent demonstrated the highest selectivity for CH_4_/N_2_, lying within 1400 to 20, followed by CO_2_/N_2_, which lies within 130 to 12. The smallest selectivity is associated with C_2_H_6_/CH_4_, which lies within 5 to 2. It should be noted that separation of CO_2_ from CH_4_ is also a highly important industrial separation for purification of natural gas [37]. Although a majority of carbon-based adsorbents are more selective toward CO_2_ over CH_4_ due to the inherent basicity of the graphene plane of carbon [36], the 3D printed carbon demonstrated an alternating behavior of selectivity; it is more selective to CH_4_ in the pressure lower than 20 kPa, above which its selectivity switches to CO_2_. Owing to such altering behavior, we did not calculate the IAST-based selectivity of CO_2_ and CH_4_.

**Figure 10:**
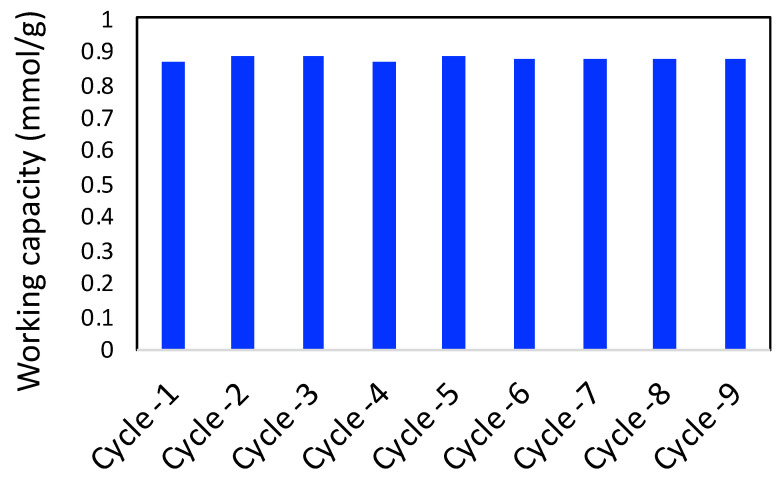
Cyclability of working capacity of CO_2_ adsorption on 3D printed porous carbon monolith.

The adsorbent material should have a good cyclability of the working capacity of the adsorbate. In this work, we have chosen CO_2_ to be the adsorbate gas and performed 10 cycles of adsorption and desorption. The working capacity was generally calculated as the difference between the adsorbed amount at 1 bar and the desorbed amount at 0.1 bar. The ten cycles of the working capacity of CO_2_ adsorption are shown in Figure 10. As observed in the figure, working capacity did not vary by more than 2–3%, suggesting a good cyclability of the adsorbent.

## 4. Conclusions

In this research, we have successfully fabricated a 3D printed carbon monolith using the direct ink method. To the best of our knowledge, it is the first report of the synthesis of 3D printed porous carbon without any sacrificial template. The commercial activated carbon was the main source of porous carbon along with poly(4-vinylphenol) and Pluronic F127 that simultaneously acted as the plasticizer and origin of mesoporosity. The adsorbent possesses a BET surface area of 400 m^2^/g and a total pore volume of 0.27 cm^3^/g. Pure component adsorption of CO_2_, CH_4_, C_2_H_6_, N_2_, CO, and H_2_ on the carbon monolith confirmed that stable adsorption capacity demonstrated the highest selectivity for CH_4_/N_2_ pair. The cyclability of working capacity of CO_2_ on this carbon confirmed that the stable structure of adsorbent does not degrade under repeated loading and unloading of adsorbates.

## Figures and Tables

**Table 1:** Fitting parameters of Sips equation.

Sips Constants	CH_4_	C_2_H_6_	CO_2_	CO	N_2_	H_2_
$a_{m}$	500	499.048	499.025	68.649	68.649	68.649
b	1.9 × 10^−4^	2.3 × 10^−4^	3.2 × 10^−5^	1.54 × 10^−6^	2.28 × 10^−6^	1.59 × 10^−7^
n	3.633	2.515	1.555	0.897	0.95	0.891

## Data Availability

The data presented in this study are available on request from the corresponding author.

## Sample Availability

Samples of the compounds are not available from the authors.
